# Heart sparing radiotherapy in breast cancer: the importance of baseline cardiac risks

**DOI:** 10.1186/s13014-020-01520-8

**Published:** 2020-05-24

**Authors:** Aurélie Gaasch, Stephan Schönecker, Cristoforo Simonetto, Markus Eidemüller, Montserrat Pazos, Daniel Reitz, Maya Rottler, Philipp Freislederer, Michael Braun, Rachel Würstlein, Nadia Harbeck, Maximilian Niyazi, Claus Belka, Stefanie Corradini

**Affiliations:** 1 Department of Radiation Oncology, University Hospital, LMU Munich, Marchioninistraße 15, 81377 Munich, Germany; 2 Institute of Radiation Medicine, Helmholtz Center Munich, Munich, Germany; 3 Red Cross Breast Centre, Munich, Germany; 4 Department of Obstetrics and Gynecology, Breast Centre, University Hospital, LMU Munich, Munich, Germany

**Keywords:** Breast cancer, Breast conserving surgery, Radiotherapy, Heart sparing, Deep inspiration breath hold (DIBH), Toxicity, Radiation-induced risk, Cardiovascular risk factors, Cardiac risk, Outcome

## Abstract

**Background:**

Patients with left-sided breast cancer have an increased risk of cardiovascular disease (CVD) after radiotherapy (RT). While the awareness of cardiac toxicity has increased enormously over the last decade, the role of individual baseline cardiac risks has not yet been systematically investigated. Aim of the present study was to evaluate the impact of baseline CVD risks on radiation-induced cardiac toxicity.

**Methods:**

Two hundred ten patients with left-sided breast cancer treated in the prospective Save-Heart Study using a deep inspiration breath-hold (DIBH) technique were analysed regarding baseline risk factors for CVD. Three frequently used prediction tools (Procam, Framingham and Reynolds score) were applied to evaluate the individual CVD risk profiles. Moreover, 10-year CVD excess absolute risks (EAR) were estimated using the individual mean heart dose (MHD) of treatment plans in free breathing (FB) and DIBH.

**Results:**

The individual baseline CVD risk factors had a strong impact on the 10-year cumulative CVD risk. The mean baseline risks of the non-diabetic cohort (*n* = 200) ranged from 3.11 to 3.58%, depending on the risk estimation tool. A large number of the non-diabetic patients had a very low 10-year CVD baseline risk of ≤1%; nevertheless, 8–9% of patients reached ≥10% baseline 10-year CVD risk. In contrast, diabetic patients (*n* = 10) had significantly higher baseline CVD risks (range: 11.76–24.23%). The mean 10-year cumulative risk (Framingham score) following RT was 3.73% using the DIBH-technique (MHD:1.42Gy) and 3.94% in FB (MHD:2.33Gy), after adding a 10-year-EAR of + 0.34%(DIBH) and + 0.55%(FB) to the baseline risks, respectively. Smoking status was one of the most important and modifiable baseline risk factors. After DIBH-RT, the 182 non-smoking patients had a mean 10-year cumulative risk of 3.55% (3.20% baseline risk, 0.35% EAR) as compared to 6.07% (5.60% baseline risk, 0.47% EAR) for the 28 smokers.

**Conclusion:**

In the present study, all CVD prediction tools showed comparable results and could easily be integrated into daily clinical practice. A systematic evaluation and screening helps to identify high-risk patients who may benefit from primary prevention. This could result in an even higher benefit than from heart-sparing irradiation techniques alone.

## Background

Multimodal breast cancer therapies have evolved rapidly over the last decades and nowadays breast cancer patients represent one of the largest survivorship groups [1]. Minimizing therapeutic morbidity has therefore become a major topic of concern.

It is well known, that the risk of developing cardiovascular disease (CVD) is significantly higher in breast cancer patients treated with radiotherapy [2]. Especially in left-sided breast cancer, the dose to the heart is approximately two or three times higher than in right-sided breast cancer [3]. Frequently, the apex of the heart is close, or even within the radiation field, resulting in a maximum dose exposure of the heart of up to > 20Gy [4]. Recently, the awareness of heart toxicity has increased enormously and new heart sparing irradiation techniques as deep inspiration breath-hold (DIBH), prone position or intensity modulated radiotherapy (IMRT) have been applied to significantly reduce the heart dose and the risk of future cardiac events [5].

Nevertheless, the role of individual baseline cardiac risks within this setting has not yet been systematically investigated in real-world cohorts. Pre-existing cardiac risk factors can further increase the risk of heart disease following radiotherapy [6]. These factors include age, history of hypertension or diabetes mellitus, elevated cholesterol levels, positive family history (myocardial infarction < 60 years), smoking habits, or individual sensitivity to late heart disease [7, 8]. However, to date, very few studies have addressed the significance and influence of baseline cardiac risk factors prior to radiotherapy in breast cancer [9–11]. The ground-breaking case-control study of Darby et al. [2] analysed the incidence of major coronary events (myocardial infarction, coronary revascularization, or death from ischemic heart disease) in 2168 women who underwent radiotherapy for breast cancer between 1958 and 2001. The mean heart dose (MHD) using elderly techniques was 6.6 Gy in left-sided breast cancer, which was significantly higher than that using modern DIBH techniques. Darby calculated a linear increase of the relative cardiovascular risk (excess relative risk, ERR) of 7.4% per Gy mean heart dose (95% confidence interval, 2.9 to 14.5%; *P* < 0.001) for the entire cohort. This fact gained wide public attention and was the beginning of the heart sparing area in modern breast radiotherapy. While most radiation oncologists are aware of an 7.4% ERR increase per Gy, there were further interesting details regarding cardiac risk factors in the study, which have not received comparable attention. Women without a history of ischemic heart disease and the presence of one or more cardiac risk factors at the time of breast cancer diagnosis (e.g. current smoker, high body-mass index, diabetes, chronic obstructive pulmonary disease) had a significantly elevated rate ratio for major coronary events of 2.60 (95% CI, 1.89 to 3.57 during the first 10 years). After taking into account dose exposure to the heart, the relative percentage increase in the rate of major coronary events per Gy was similar for women with and those without cardiac risk factors, leaving the baseline cardiac risks as the most important predictor for absolute 10-year CVD risks.

Aim of the present study was to systematically evaluate cardiovascular risk factors and their influence on cardiovascular risk estimates in a cohort of left-sided breast cancer patients treated with modern radiotherapy techniques using a DIBH technique in clinical practice.

## Methods

All patients were enrolled in the prospective Save-Heart study for deep-inspiration breath hold (DIBH) radiotherapy in left-sided breast cancer. The study was approved by the ethics committee of the LMU medical faculty (13.09.2016, No. 355–16) and registered in the Clinical Trials Register (DRKS-ID: DRKS00011213). Inclusion criteria were informed consent, left-sided breast cancer or carcinoma in-situ and patient compliance for DIBH (ability of breath-hold for at least 20 s) [12].

From October 2016 to January 2019, a total of 352 patients were enrolled in the present study and gave informed consent. An individual cardiovascular risk profile assessment was performed for all eligible patients. For this purpose, a specific questionnaire was elaborated to record all baseline cardiovascular risk factors. The evaluated parameters included smoking behaviour, history of diabetes mellitus, antihypertensive therapy and family history of cardiovascular disease. If available, CRP and cholesterol levels (LDL, HDL, triglycerides) were reported. Patients with prior cardiac events or missing blood values were excluded from this analysis. All patients were treated using surface-guided DIBH as described elsewhere [13].

Patients with diabetes mellitus were analysed separately, as cardiovascular risk prediction is more challenging in patients with diabetes. Most CVD risk prediction tools have been developed in the general population and are likely to underestimate the cardiovascular risk in patients with diabetes [14]. Nevertheless, diabetes-specific risk scores were used to estimate cardiovascular risks in individuals with diabetes.

For the analysis of the baseline cardiac risk scores, three different clinically used risk scores were applied: the Procam score, the Framingham score and the Reynolds score. Various risk scores for the assessment of cardiovascular risks were used, to compare the results and evaluate their complementary or additive value in risk estimation in clinical practice.

The Procam score calculates the risk of major coronary events (sudden cardiac death or myocardial infarction) over the next 10 years, based on cholesterol levels (HDL, LDL and Triglycerides), gender, age, systolic blood pressure, smoking habits, family history, and diabetes [15]. The Procam coronary risk score was derived from data of the Prospective Cardiovascular Münster (PROCAM) study in Germany, using data from 18,460 men and 8515 women who were recruited from 1978 to 1995 and had a mean follow-up period of 12 ± 6 years. In the Save-Heart study, the Procam score was routinely applied to assess cardiovascular risks. Each patient with an estimated risk of > 10% was informed and counselled regarding primary prevention.

The Framingham score estimates the 10-year risk of any cardiovascular event (coronary heart disease, cardiovascular disease, cerebrovascular events, peripheral artery disease and heart failure). The score was developed based on findings from the longitudinal Framingham Heart Study on residents of the city of Framingham, Massachusetts, USA, since 1948. It takes into account the following parameters: gender, age, smoking habit, diabetes, total cholesterol, HDL cholesterol, systolic blood pressure, and antihypertensive medication [16].

Similarly, the Reynolds score predicts the 10-year risk of cardiovascular events (myocardial infarction, ischemic stroke, coronary revascularization, and cardiovascular death). The Reynolds risk score for women was developed and validated using data from 24,558 initially healthy American women who were followed over a 10-year period. The score uses the variables: gender, age, smoking habit, diabetes, systolic blood pressure, total cholesterol, HDL cholesterol, (high sensitivity) CRP, and family history [17].

For each patient, all three risk scores were used to estimate the individual baseline cardiovascular risk before radiotherapy. Univariate ANOVA with repeated measures was used for differences between the three risk scores. Correction of Holms-Bonferroni regarding multiple testing was applied. For continuous data the Wilcoxon Signed-Rank test was used for paired samples and the Mann-Whitney U-test for independent samples. Significance level of *p* = 0.05 was applied for all statistical analyses. For calculation of the 10-year CVD excess absolute risk (EAR), the relative increase of baseline risks after radiotherapy was calculated using the mean heart dose (MHD) according to Darby et al. [2] This calculation assumes a linear increase of the relative cardiovascular risk (excess relative risk, ERR) of 7.4% per Gray mean heart dose. The MHD was derived from dose-volume-histograms of treatment plans in free breathing and DIBH. Thereafter, the impact of radiotherapy was analysed using the baseline risk of all scores to define the absolute 10-year risk for cardiovascular events after radiotherapy as follows:

$$ Cumulative\kern0.5em risk= baseline\kern0.5em risk+ EAR. $$

$$ EAR= ERR\ast baseline\kern0.5em risk= MHD\ast {\delta}_{Darby}\ast baseline\kern0.5em risk;\cdot \left({\delta}_{Darby}=0.074{Gy}^{-1}\right). $$

## Results

After exclusion of patients with a history of CVD events, the individual cardiovascular baseline risk scores of 200 non-diabetic and 10 diabetic breast cancer patients were calculated based on individual cardiovascular risk factors. An overview of the evaluated cardiovascular risk factors is given in Table 1. Overall, a large number of patients was estimated to have a very low 10-year CVD baseline risk of ≤1% according to all three prediction tools: 45% using the Procam score (90/200 patients), 46% using the Framingham score (92/200 patients) and 48% as predicted by the Reynolds score (96/200 patients) (Table 2). In contrast, only 8–9% of the non-diabetic patients reached high risk scores of ≥10% baseline 10-year CVD risk. The mean risks of the non-diabetic cohort ranged from 3.11% (±5.14, 95% CI: 2.39–3.83%, Procam score), 3.39% (±3.67, 95% CI: 2.88–3.90%, Framingham score) to 3.58% (±4.70, 95% CI: 2.92–4.23%, Reynolds score).

**Table 1:** Cardiovascular risk factors of 210 left-sided breast cancer patients. FB: free-breathing, DIBH: deep inspiration breath-hold

		Non-diabetic patients (*n* = 200)		Diabetic patients (*n* = 10)	
Age at diagnosis (years)	< 40	16	(8.0%)	0	
	40–49	32	(16.0%)	2	(20%)
	50–59	73	(36.5%)	2	(20%)
	60–69	45	(22.5%)	5	(50%)
	70–79	33	(16.5%)	1	(10%)
	≥80	1	(0.5%)	0	
	*Mean*	*57*		*60*	
Smoking habit	positive	26	(13.0%)	2	(20%)
	negative	174	(87.0%)	8	(80%)
Family history	positive	27	(13.5%)	1	(10%)
	negative	173	(86.5%)	9	(90%)
LDL (mg/dL)	< 100	37	(18.5%)	3	(30%)
	100–149	111	(55.5%)	6	(60%)
	150–199	43	(21.5%)	0	
	> 200	9	(4.5%)	1	(10%)
	*Mean*	*130*		*115*	
HDL (mg/dL)	< 50	20	(10%)	4	(40%)
	50–99	169	(84.5%)	6	(60%)
	> 100	11	(5.5%)	0	
	*Mean*	*69*		*57*	
Triglycerides (mg/dL)	< 50	5	(2.5%)	1	(10%)
	50–99	84	(42.0%)	2	(20%)
	100–149	62	(31.0%)	1	(10%)
	150–199	23	(11.5%)	1	(10%)
	200–249	19	(9.5%)	3	(30%)
	> 250	7	(3.5%)	2	(20%)
	*Mean*	*122*		*171*	
Total Cholesterol (mg/dL)	100–149	3	(1.5%)	1	(10%)
	150–199	54	(27.0%)	5	(50%)
	200–249	97	(48.5%)	3	(30%)
	250–299	34	(17.0%)	1	(10%)
	> 300	12	(6.0%)	0	
	*Mean*	*223*		*203*	
(hs) CRP (mg/dL)	< 0.1	65	(32.5%)	2	(20%)
	0.1–0.5	115	(57.5%)	6	(60%)
	> 0.5	20	(10.0%)	2	(20%)
	*Mean*	*0.23*		*0.55*	
Mean heart dose DIBH (Gy)	< 1.0	37	(18.5%)	0	
	1.0–1.9	142	(71.0%)	6	(60%)
	2.0–4.9	20	(10.0%)	4	(40%)
	> 5.0	1	(0.5%)	0	
	*Mean*	*1.42*		*1.85*	
	*95% CI*	*1.34–1.50*		*1.47–2.24*	
Mean heart dose FB (Gy)	< 1.0	10	(5.0%)	0	
	1.0–1.9	87	(43.5%)	1	(10%)
	2.0–4.9	97	(48.5%)	9	(90%)
	> 5.0	6	(3.0%)	*0*	
	*Mean*	*2.33*		*2.86*	
	*95% CI*	*2.17–2.49*		*2.26–3.47*

**Table 2:** 10-year cardiovascular disease (CVD) baseline risk scores of 210 left-sided breast cancer patients

10-year-CVD risk (%)	Non-Diabetic patients (*n* = 200)		Diabetic patients (*n* = 10)	
Procam Score				
≤1	90	(45%)	1	(10%)
1.1–4.9	76	(38%)	3	(30%)
5–9.9	16	(8%)	1	(10%)
10–19.9	15	(7.5%)	3	(30%)
> 20	3	(1.5%)	2	(20%)
*mean*	*3.11*		*11.76*	
*95% CI*	*2.39–3.83*		*2.86–20.65*	
Framingham Score				
≤1	92	(46%)	0	
1.1–4.9	53	(26.5%)	1	(10%)
5–9.9	39	(19.5%)	2	(20%)
10–19.9	15	(7.5%)	2	(20%)
> 20	1	(0.5%)	5	(50%)
*mean*	*3.39*		*24.23*	
*95% CI*	*2.88–3.90*		*13.79–34.67*	
Reynolds Score				
≤1	96	(48%)	1	(10%)
1.1–4.9	53	(26.5%)	4	(40%)
5–9.9	35	(17.5%)	0	
10–19.9	13	(6.5%)	3	(30%)
> 20	3	(1.5%)	2	(20%)
*mean*	*3.58*		*10.66*	
*95% CI*	*2.92–4.23*		*3.89–17.43*	

The same analysis was performed for the 10 diabetic patients included in the present analysis (Table 2). These patients had significantly higher baseline CVD risks (11.76 ± 12.43% Procam score, 95% CI: 2.86–20.65, 24.23 ± 14.59% Framingham score, 95% CI: 13.79–34.67, 10.66 ± 9.46% Reynolds score, 95% CI: 3.89–17.43%) as compared to non-diabetic patients (for each pair *p* < 0.01). Only one diabetic patient had a calculated risk of ≤1% using the Procam and Reynolds CVD prediction and no patient using the Framingham estimate (Table 2).

**Fig. 1:**
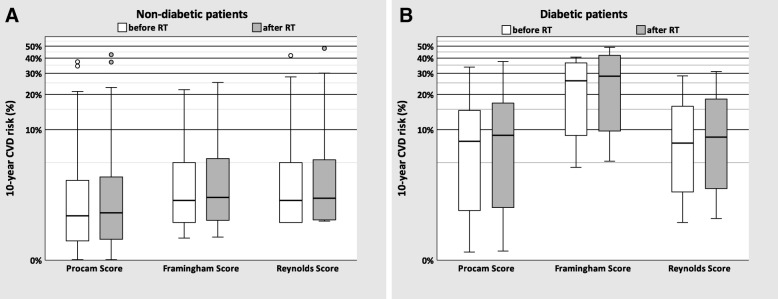
Box plot of mean 10-year baseline cardiovascular disease (CVD) risk of 200 non-diabetic patients (**a**) and 10 diabetic patients (**b**) as calculated by different risk estimation tools (white bars); and absolute mean 10-year cumulative CVD risk after left-sided breast radiotherapy in DIBH after taking into account a linear increase of 7.4% per Gy mean heart dose (grey bars). In the box plots, the boundary of the box closest to zero indicates the 25th percentile, a black line within the box marks the median and the boundary of the box farthest from zero indicates the 75 th percentile. Whiskers above and below the box indicate the 1.5 interquartile range (IQR). Points above and below the whiskers indicate outliers outside the 1.5 IQR (1.5–3 IQR) and > 3 IQR

To estimate the impact of baseline CVD risk estimates on the absolute 10-year risk of cardiovascular events after heart-sparing DIBH-radiotherapy, the individual mean heart doses were taken into account. The mean absolute 10-year risk increase (EAR) following DIBH-RT in non-diabetic patients was + 0.30% (±0.55; with a maximum increase of + 5.33%; Procam score, 95% CI: 0.23–0.38%), + 0.34% (±0.42; with a maximum increase of + 3.14%, Framingham score, 95% CI: 0.28–0.40%) and + 0.37% (±0.56; with a maximum increase of + 6.00%, Reynolds score, 95% CI: 0.29–0.45%) (Fig. 1, Table 3). In other words, the absolute cumulative 10-year CVD risk rose to 3.41% (±5.66, Procam score, 95% CI: 2.62–4.20%), 3.73% (±4.04, Framingham score, 95% CI: 3.17–4.30%) and 3.95% (±5.22, Reynolds score, 95% CI: 3.22–4.67%). To give an order of magnitude, 18 of 200 patients evaluated with the Procam score had an absolute baseline risk of ≥10%, including 3 patients with a risk of ≥20% to encounter a sudden cardiac death or myocardial infarction in the 10 years following radiotherapy. After taking into account the radiation-induced risk increase due to adjuvant DIBH-RT, two low-risk patients moved to this high risk group.

**Table 3:** 10-year cardiovascular cumulative risk of 210 left-sided breast cancer patients following radiotherapy after taking into account an increase of 7.4% per Gy in mean heart dose of the individual treatment plans. FB: free-breathing, DIBH: deep inspiration breath-hold, EAR: excess absolute risk

	Non-Diabetic patients (*n* = 200)		Diabetic patients (*n* = 10)		Entire cohort (*n* = 210)	
	Following FB-RT	Following DIBH-RT	Following FB-RT	Following DIBH-RT	Following FB-RT	Following DIBH-RT
Procam Score						
Baseline risk	3.11%		11.76%		3.52%	
Mean EAR	+ 0.50%	+ 0.30%	+ 1.92%	+ 1.52%	+ 0.57%	+ 0.36%
Mean cumulative risk	3.61%	3.41%	13.68%	13.28%	4.09%	3.88%
Framingham Score						
Baseline risk	3.39%		24.23%		4.38%	
Mean EAR	+ 0.55%	+ 0.34%	+ 4.64%	+ 3.37%	+ 0.75%	+ 0.49%
Mean cumulative risk	3.94%	3.73%	28.87%	27.60%	5.13%	4.87%
Reynolds Score						
Baseline risk	3.58%		10.66%		3.91%	
Mean EAR	+ 0.60%	+ 0.37%	+ 1.88%	+ 1.47%	+ 0.67%	+ 0.42%
Mean cumulative risk	4.18%	3.95%	12.54%	12.13%	4.58%	4.33%

**Fig. 2:**
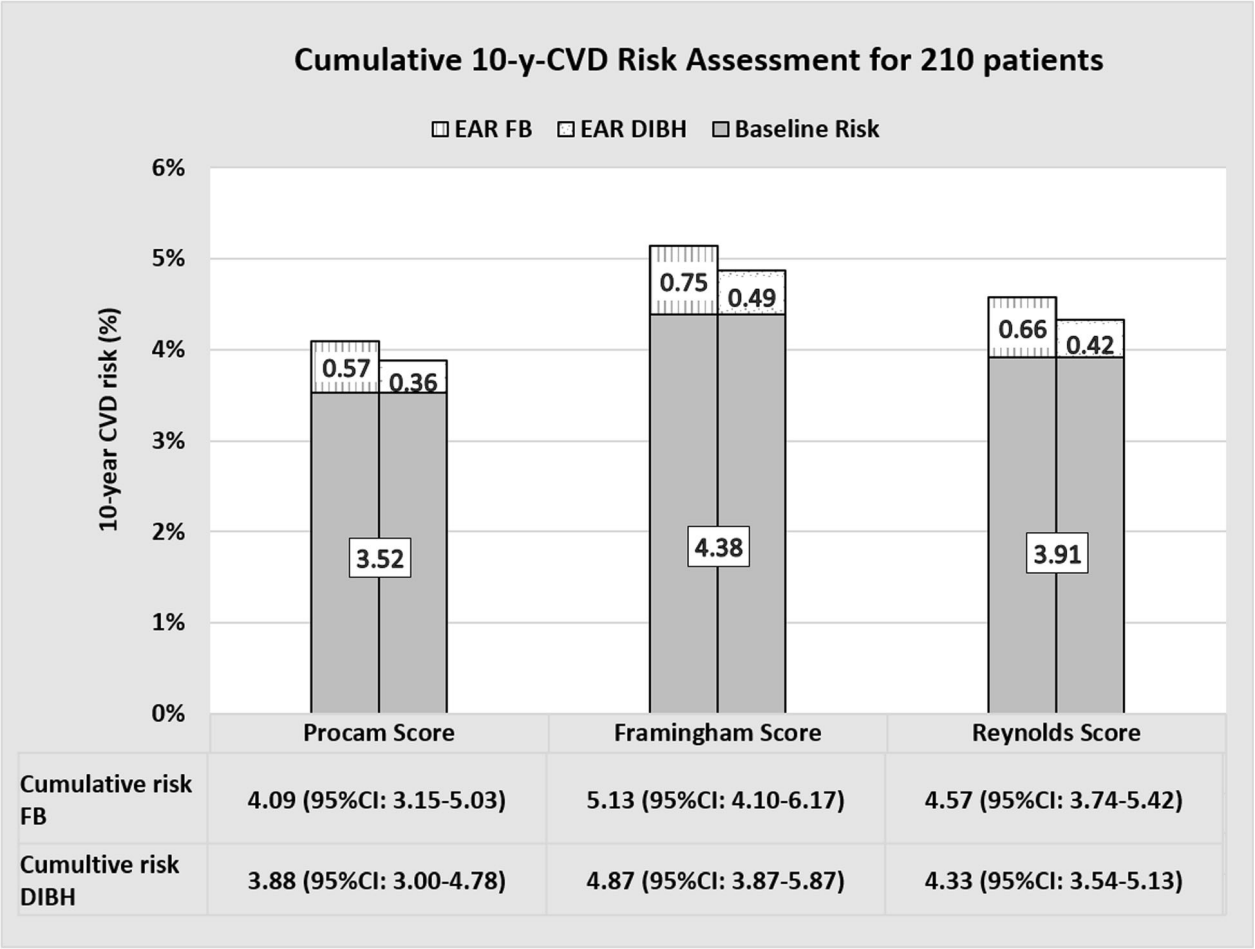
Mean cumulative 10-year CVD risk of 210 patients after FB−/DIBH-RT as calculated by 3 different risk calculators. FB: free-breathing, DIBH: deep inspiration breath-hold, EAR: excess absolute risk

In the Save-Heart Study, all patients were irradiated using a heart-sparing DIBH technique. Nevertheless, for every study patient, an additional RT plan in free-breathing (FB) was calculated to analyse the dosimetric benefits of the DIBH-technique. This dual treatment planning allowed to compare the cardiovascular risks of DIBH and FB (Fig. 2). The excess relative risk (ERR) was 11% (±5, 95% CI: 0.10–0.11%) following DIBH-RT and 17% (±9, 95% CI: 0.16–0.19%) following FB-RT, corresponding to a relative cardiac risk increase of + 64.7% for FB as compared to DIBH. The cumulative absolute 10-year CVD risks after left-sided breast irradiation are listed in Table 3.

**Fig. 3:**
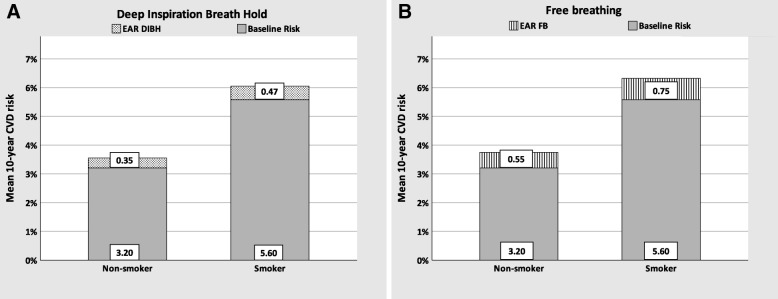
Mean cumulative 10-year CVD risk estimates of the entire cohort regarding their smoking habit (28 smokers, 182 non-smokers) after DIBH-RT (**a**) and FB-RT using Procam 10-year CVD risk score (**b**)

Furthermore, the patient cohort was analysed with regard to their smoking habits and baseline risks using the Procam score (Fig. 3). A total of 28 active smokers were identified in the entire cohort of 210 patients with left-sided breast cancer. Regarding 10-year cumulative risk following DIBH-RT, smokers had a risk of 6.07% (5.60% baseline risk, + 0.47% EAR, 95% CI: 2.21–9.89%), in contrast to 3.55% (3.20% baseline risk + 0.35% EAR, 95% CI: 2.69–4.41%) in non-smoking patients (diabetic and non-diabetic). If patients had been treated with a FB technique, smokers would have an estimated 10-year cumulative risk of 6.35% (5.60% baseline risk + 0.75% EAR, 95% CI: 2.38–10.27%), while non-smokers have a lower CVD risk of 3.75% (3.20% baseline risk + 0.55% EAR, 95% CI: 2.84–4.66%).

## Discussion

During the last decade, the awareness regarding heart toxicity due to irradiation of left-sided breast cancer has increased enormously. The radiation-related mortality risks from heart disease may not occur immediately after dose exposure, but may persist for many years and increase over time [18]. As a result, new heart sparing RT techniques have been introduced into clinical practice that can significantly reduce dose exposure of the heart in order to prevent cardiac morbidity. Nevertheless, the role of individual baseline cardiac risk factors has never been evaluated systematically within this context. There is only some evidence for single CVD risk factors, such as smoking or hypertension. The retrospective study of Hooning et al. [11] found that a more than additive effect of smoking and cardiac radiation exposure leads to significantly increased rates of fatal myocardial infarction (HR = 3.04, 95%CI: 2.03–4.55 vs non-smokers without radiation) in patients treated with breast irradiation. Similarly, Harris et al. [9] found increased rates of coronary artery disease in patients receiving left-sided radiation that had a history of hypertension (HR = 11.4, 95%CI: 5.0–26.2 vs no high blood pressure with right-sided radiation).

As reported in the present study, the main factors influencing 10-year CVD excess absolute risk (EAR) were the individual baseline risks of patients. In this cohort of 200 non-diabetic patients the mean baseline CVD risk ranged from 3.11 to 3.58%, depending on which risk estimation tool was used. Radiation exposure leads to an ERR of 11% following DIBH-RT and 17% following FB-RT. To give an order of magnitude, this corresponds to a mean 10-year EAR of 0.30–0.37% in DIBH and 0.50–0.60% in FB.

While the community focusses on how to use modern RT techniques to minimize heart exposure, aim of the present study was to raise the awareness for baseline cardiac risk factors and their importance within this setting. The present study used different clinically applicable risk scores (Procam Score, Framingham Score and Reynolds Score), which all showed comparable results and can easily be integrated in daily clinical routine in radiation oncology. If we put all this effort in minimizing the radiation dose to the heart, a systematic evaluation and counselling regarding CVD risk factors appears feasible and could further help to lower cardiac burden. As shown in the present study, this could result in an even higher benefit as from heart-sparing irradiation techniques alone. Obviously, the authors are advocates of heart-sparing breast cancer radiotherapy techniques and recommend to routinely use them.

Smoking was one of the most important and modifiable risk factors for CVD in the present study. After estimating the baseline risks of the 28 smoking patients using the Procam score, the estimates improved if the smoking status was set to non-smoking (5.60% vs 2.46%), which corresponds to a relative decrease of − 56% (*p* < 0.01). This effect was much more pronounced than the impact of the different radiotherapy techniques on 10-year cumulative risk (FB vs DIBH: 6.32% vs 6.05%, corresponding to a relative decrease of − 4.3%). This fact is also known from several other studies, where smoking cessation significantly reduced the risk of myocardial infarction by about 65% [19]. Moreover, primary care research suggests, that simple counselling of the patient can help substantially increase smoking cessation rates [20]. Therefore, it seems advisable to make smoking cessation counselling a standard component of RT consultation, where all breast cancer patients should be screened for their smoking status, informed about the health benefits and supported with help in smoking cessation [21]. In the interdisciplinary tumorboard of the LMU Breast centre, smoking cessation is already routinely recommended, as it not only reduces the radiation-induced CVD risk, but also the risk of secondary lung cancer [12].

Overall, patients with multiple cardiovascular risk factors, or patients with diabetes or metabolic syndrome are at high risk for subsequent CVD events [22]. It is important to identify these patients prior to RT and develop a CVD risk reduction plan, accordingly. It will be necessary to educate these patients about the health benefits and importance of cardiac events prevention. Therapeutic lifestyle changes, like healthy nutrition, weight loss, smoking cessation, and increased physical activity can significantly reduce cardiac toxicity after radiation exposure and the patient can actively contribute to this. In addition, adjunctive drug therapies like antihypertensive medication or statins may be appropriate measures regarding hypertension and dyslipidemia [23, 24]. This detailed primary CVD prevention can be performed by the primary care providers, if elevated CVD risk scores are detected during RT screening. Moreover, young patients with multiple CVD risk factors (diabetes, hypertension, smoking) can still reach low 10-year cardiovascular risk score levels due to their young age. In these patients, it seems advisable to take preventive measures, especially if they have a good cancer prognosis [12].

The different prediction tools were calibrated in different geographical regions and in diverse patient and population cohorts [25]. Nevertheless, our analysis showed that all three risk prediction tools report comparable results in non-diabetic patients. They showed a low individual variability and each of the scores seems feasible to assess the baseline cardiac risk of RT patients. The good news is, that the vast majority of non-diabetic patients presented with a low CVD baseline risk (45–48% had a 10-year risk ≤1%) and did not need any measures. However, 8–9% of the present 200 patient cohort did reach higher risk scores of > 10% in 10-year baseline CVD risk. In consequence, their relative increase through incidental heart irradiation reaches a higher absolute value. This subgroup of patients will benefit substantially from a heart-sparing irradiation technique [12].

Patients with diabetes were analysed separately, as most CVD risk prediction tools were developed in the general population and are likely to underestimate the cardiovascular risk in patients with diabetes [13]. Usually, if diabetes is taken into account, patients are predicted with a significant 10-year risk for CVD (> 10%). As shown in the present study, diabetes-specific risk estimates differed severely regarding the results of the different prediction tools, which shows the limitations of risk prediction in patients with diabetes. Both, the Procam and Reynolds scores seem to assess the cardiovascular risk similarly for diabetic patients, but the Framingham score estimated a twofold higher risk. Indeed, the Framingham CVD model was poorly calibrated for the endpoint of major CVD, as it was developed for the broader endpoint of total CVD (coronary insufficiency, angina, peripheral artery disease, TIA) [26].

A limitation of the present study could be the use of the mean heart dose for risk calculation. The original study of Darby et al. [2] did not use dose parameters from real treatment plans, but estimates of cardiac dose based on reconstructions of patients with standard anatomy and common radiotherapy regimens. As known from several current reviews and recommendations addressing heart dose constraints and heart-sparing techniques [27, 28], the dose to cardiac subvolumes, such as the left ventricle or the left descending arteries should be reported in addition to mean heart dose. Nevertheless, the mean heart dose was used in the present study to apply the above-mentioned risk calculation models.

In conclusion, risk estimates of baseline cardiac risks should be included in clinical practice. In high risk patients, primary prevention with counselling or pharmacotherapy interventions could provide substantial immediate and long-term health benefits. Moreover, if these procedures are accompanied by maximum cardiac protection during breast radiotherapy, cardiac morbidity could be substantially reduced. The approach to use clinically available risk prediction tools is a cost-effective intervention which can easily be adopted during routine patient care. It would be favourable in the near future, to include dose parameters of heart exposure to further individualize risk prediction. Nevertheless, such risk modelling calculators are not yet broadly available [29]. Key to successful implementation in clinical practice is the awareness of radiation oncologists on the importance of baseline CVD risks. It is important to minimize the burden for cardiac toxicity and radiation oncologists must come to see their role in promoting primary or secondary prevention within this setting.

## Data Availability

Not applicable.

## Abbreviations

CVD: Cardiovascular disease
CI: Confidence interval
CRP: c-reactive protein
DIBH: Deep inspiration breath-hold
EAR: Excess absolute risks
ERR: Excess relative risk
FB: Free breathing
Gy: Gray
HR: Hazard ratio
HDL: High density lipoprotein
IMRT: Intensity modulated radiation therapy
IQR: Interquartile range
LDL: Low density lipoprotein
MHD: Mean heart dose
TIA: Transient ischemic attack
